# Population genetic and biophysical evidences reveal that purifying selection shapes the genetic landscape of *Plasmodium falciparum* RH ligands in Chhattisgarh and West Bengal, India

**DOI:** 10.1186/s12936-020-03433-z

**Published:** 2020-10-14

**Authors:** Sharmistha Ghoshal, Pramita Chowdhury, Sanhita Ray, Mitashree Mitra, Sumana Datta Kanjilal, Srikanta Sen, Anjan Kr. Dasgupta, Sanghamitra Sengupta

**Affiliations:** 1 Department of Biochemistry, University of Calcutta, 35, Ballygunge Circular Road, Kolkata, 700 019 West Bengal India; 2 School of Studies in Anthropology. Pt, Ravishankar Shukla University, Raipur, 492010 Chhattisgarh India; 3 Department of Pediatric Medicine, Institute of Post Graduate Medical Education & Research, Kolkata, West Bengal India; 4 Mitra Tower, Lake Town, Block-A, Kolkata, 700 089 India

**Keywords:** *Plasmodium falciparum*, Malaria, PfRH, Polymorphism, Selection, Vaccine

## Abstract

**Background:**

Reticulocyte binding protein-like homologs (RHs) are currently being evaluated as anti-erythrocytic stage vaccine targets against *Plasmodium falciparum* malaria. Present study explores the possible evolutionary drivers shaping the genetic organization of *Pfrh*s in Indian parasite population. It simultaneously evaluates a putative gain-of-function variant of PfRH5, a keystone member of PfRH family.

**Methods:**

Receptor binding regions of *Pfrh1*, *Pfrh2a/b*, *Pfrh4* and whole *Pfrh5* were amplified using blood samples of *P. falciparum* malaria patients from Chhattisgarh and West Bengal and sequenced. Assembled sequences were analysed using MEGA7 and DnaSPv6. Binding affinities of recombinant PfRH5 proteins with basigin (BSG) were compared using in silico (CHARMM and AUTODOCK) and in vitro (Circular dichroism, fluorescence spectroscopy and isothermal titration calorimetry) methods.

**Results:**

*Pfrh1* (0.5), *Pfrh2a/b (*0.875), *Pfrh4* (0.667) and *Pfrh5* (0.778) sequence changes corresponded to low frequency (< 0.05) variants which resulted in an overall negative Tajima’s D. Since mismatch distribution of none of the *Pfrh* loci corroborated with the model of demographic expansion, a possible role of natural selection formulating *Pfrh* sequence diversity was investigated. Among the 5 members, *Pfrh5* displayed very high dN/dS (5.7) ratio. Nevertheless, the model of selective sweep due to presence of any advantageous substitutions could not be invoked as polymorphic nonsynonymous sites (17/18) for *Pfrh5* exceeded significantly over the divergent (62/86) ones (p = 0.0436). The majority of extant PfRH5 sequences (52/83) differed from the reference Pf3D7 allele by a single amino acid mismatch (C203Y). This non-conservative alteration was predicted to lower the total interaction energy of that PfRH5_variant_ with BSG, compared to PfRH5_3D7_. Biophysical evidences validated the proposition that PfRH5_variant_ formed a more stable complex with BSG. Thermodynamic association constant for interaction of BSG with PfRH5_variant_ was also found to be higher (Ka_variant_ = 3.63E6 ± 2.02E6 M^−1^ and Ka_3D7_ = 1.31E6 ± 1.21E6 M^−1^).

**Conclusions:**

Together, the study indicates that the genetic architecture of *Pfrh*s is principally shaped by purifying selection. The most abundant and ubiquitous PfRH5 variant harbouring 203Y, exhibits a greater affinity for BSG compared to PfRH5_3D7_ possessing 203C allele. The study underscores the importance of selecting the functional allele that best represents circulating strains in natural parasite populations as vaccine targets.

## Background

In spite of significant advancement in controlling malaria mortality, the disease particularly the one caused by *Plasmodium falciparum* remains a global health concern. Intervention of malaria is typically based on two regimens those include (a) control of anopheles mosquito vector by the use of insecticidals and (b) treatment of patients using anti-malarial drugs. Drug resistant parasites arising due to wide and indiscriminate deployment of chloroquine, sulfadoxine-pyrimethamine and mefloquine based mono-therapies, is causing resurgence of disease in many parts of the world [1–3]. The extraordinary evolutionary plasticity of *P. falciparum* has not even spared artemisinin (ART) based combination therapy, which currently serves as the first-line treatment for multidrug-resistant *P. falciparum* malaria [4]. Growing incidences of resistance to ART and its derivatives in Africa and South-East Asia are threatening the sustainability of current status of malaria control [4]. Before ART is lost to parasite’s genetic adaptability, development of an efficacious vaccine and its widespread application in malarious regions are necessary to deal with rebounding malaria.

Asexual blood-stage of parasite life cycle determines the pathogenesis as well as clinical outcome of *P. falciparum* malaria. Blood stage infection begins with merozoite attachment onto RBC surface and entry inside; and culminates into intra-erythrocytic parasite replication followed by RBC rupture and induction of pro-inflammatory responses in hosts. Merozoite surface proteins that are targets of naturally acquired immunity in individuals with malaria are believed to serve as anti-erythrocytic stage vaccine candidates. Epidemiological studies have also demonstrated the feasibility of such vaccines by presenting the evidences that people living in malaria endemic areas can gradually acquire immunity against both severe malaria and clinical malaria [5, 6].

During the multi-step progression of RBC invasion, direct attachment of merozoites with erythrocytes is mediated by two protein families: *P. falciparum* reticulocyte binding protein-like homolog (PfRH) and erythrocyte binding antigen (PfEBA). Except PfRH5, both classes of ligands are transmembrane proteins which are employed redundantly by parasites for successful RBC invasion [7]. PfRH protein family is composed of five members namely PfRH1, PfRH2a, PfRH2b, PfRH4 and PfRH5. PfRH members do not share much sequence homology, although PfRH2 and PfRH5 are assumed to adopt a similar ‘Kite-like’ conformation with receptor binding sites as deduced by homology modelling [8, 9]. Although the identity of the other PfRH receptors remains to be established, complement receptor 1 (CR1) and basigin (BSG), have been shown to function as the erythrocyte receptors for PfRh4 and PfRh5, respectively [7, 8, 10]. In an effort to develop a blood-stage vaccine, *P. falciparum* 3D7 growth was shown to be inhibited by antibodies from rabbits immunized with a combination of EBA-175, PfRh2a/b, and PfRh4 [11]. Furthermore, expression and polymorphism analyses of parasite field isolates reveal PfRh2a, PfRh5 and EBA-181 to be key players of invasion pathway [10].

Existence of geographical variations in the invasion ligands suggests that the idea of one-size-fits-all type of vaccine needs to be revisited and emphasizes on the necessity of genetic and functional assessments of vaccine candidates. On this note and given the unequivocal importance of PfRH ligands in erythrocyte invasion, the present study takes an account of the genetic diversity of all 5 PfRH ligands in the *P. falciparum* field isolates from two malaria-prone regions of India, one of the large reservoirs of *P. falciparum* strains. It has been recently shown that PfRH5-BSG binding is essential for parasite’s RBC invasion as this results in an bridge between parasite and erythrocyte through which invasion elements flow [12]. Therefore, in addition to dissecting the factors responsible for extant pattern of sequence divergence of *Pfrh*s, the functional capacity of one ubiquitously observed PfRH5 variant (C203Y) has been analysed with regard to its binding with RBC receptor BSG. Altogether the knowledge accrued from this study will be useful to comprehend parasite’s evolutionary dynamics under present day drug regimens and unfold the mechanism of genetic adaptability of this important human pathogen.

## Methods

### Study area, sample collection and DNA extraction

Stored genomic DNAs previously isolated form peripheral blood samples collected from *P. falciparum* malaria infected patients admitted in Calcutta National Medical College and Hospital, Kolkata, in the year 2010 were utilized in this study [13]. Kolkata is the capital of West Bengal. Almost 10% of the total malaria cases in India are accounted in West Bengal [14]. Peripheral venous blood samples were also collected from Surguja located in Chhattisgarh, central India, during a period of 2010–2013. Surguja, incidentally a tribal dominated district, is situated in the forested area of Chhattisgarh [15]. Due to its distinct ecological and geographical conditions, Chhattisgarh contributes to ~ 12% of total malaria burden and carries the highest share of deaths (17%) in India [16]. Only blood samples from *P. falciparum* malaria positive patients confirmed by rapid diagnostic tests based on dual-Antigen and/or Giemsa-stained thick and thin smears were selected for this study [15]. Individuals suffering from co-infection with *Plasmodium vivax* and pregnant women were excluded. Additionally, the study excluded the patients suffering from chronic or severe disease conditions, such as cardiac, renal or hepatic diseases, G6PD deficiency, typhoid, measles, acute lower respiratory tract infection, bacteraemia, severe diarrhoea with dehydration, sickle cell anaemia, AIDS and cancer. Genomic DNA was isolated from the blood sample of *P. falciparum* malaria infected patients using QIAamp DNA Blood Midi Kit (Qiagen, Hilden, Germany) according to manufacturer’s protocol.

### Polymerase chain reaction, purification of PCR amplicons and sequencing of PfRHs

**Fig. 1:**
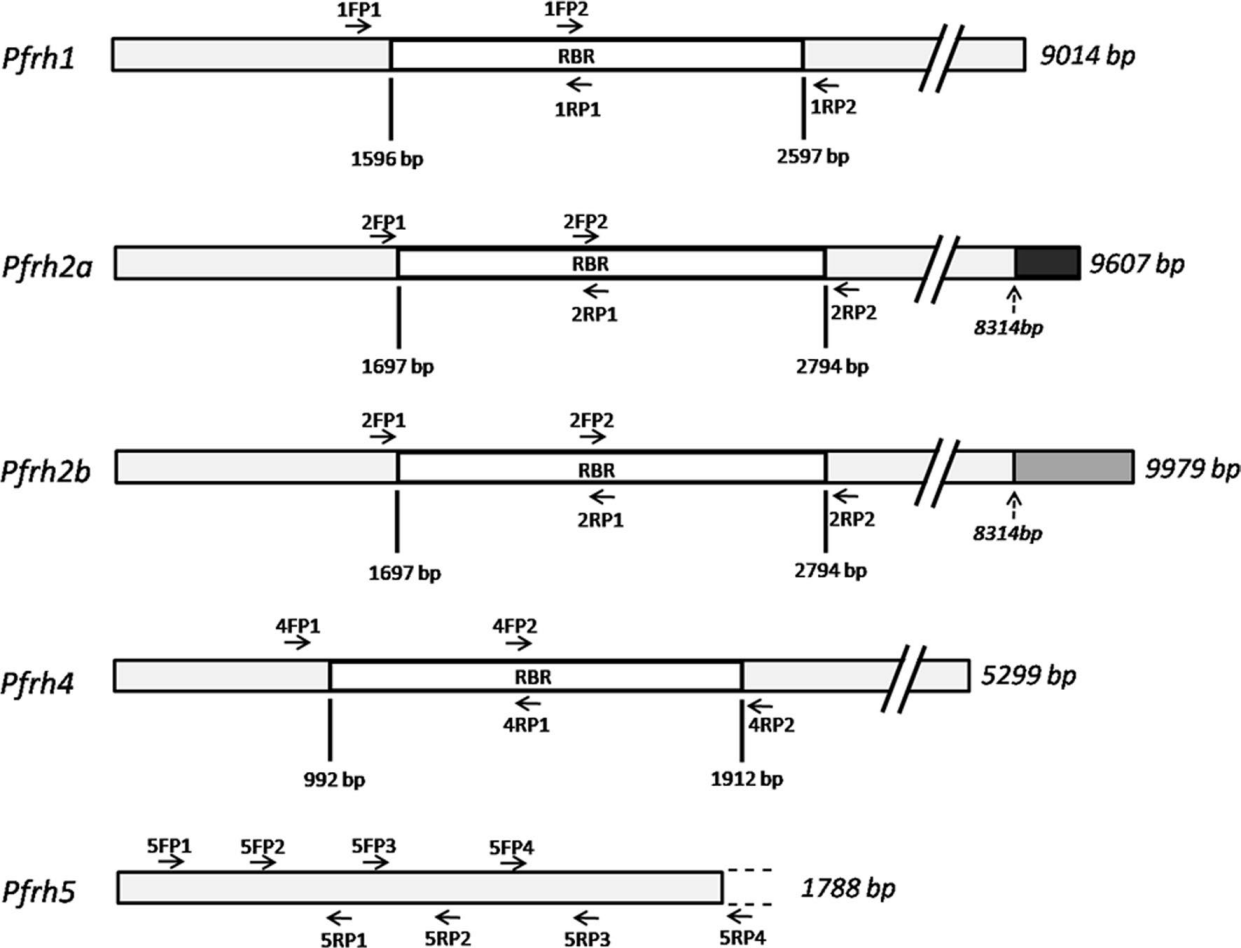
Schematic representation of genes encoding PfRH ligands. The regions subjected to sequence analysis were shown. Regions mentioned as RBR signified receptor binding region. Solid arrows represented positions of the primers used to amplify parasite genomic DNA. Arrows with broken line indicated the non-identical regions of *Pfrh2a* and *Pfrh2b.* Sequences corresponding to RBRs were analysed for *Pfrh1*, *Pfrh2a/b* and *Pfrh4*, while the entire coding region of *Pfrh5* was subjected to sequence analysis

Oligonucleotide primers for polymerase chain reaction and sequencing of genes encoding PfRHs were designed by retrieving reference sequences of *P. falciparum* 3D7 from PlasmoDB (Fig. 1) [17]. Receptor binding sites of PfRH1 and PfRH4 were amplified using two pairs of overlapping primers for each [18–20]. PfRH2a and PfRH2b possess indistinguishable ecto-domain containing the putative receptor binding site (495-860 amino acid region of PfRH2a/b in *Pf*3D7) [18, 19]. This identical receptor binding site of PfRH2a/b was considered for primer designing and amplification. An attempt was made to amplify the whole PfRH5 gene using four overlapping primer pairs. The primer sequences as well as cycling conditions were described in the Additional file 1: Table S1. Amplification of target regions were performed in 15μL reaction mixtures containing 1 U of GoTaq^®^ Flexi DNA polymerase (Promega), 0.2 mM dNTP, 1.5 mM MgCl_2_, and 0.4 μM of each primer using a GeneAmp^®^ PCR System 9700 (Applied Biosystems). UV transillumination on gel documentation system (Biostep) was used to visualize the PCR products preceded by electrophoresis on 2% agarose gel (Promega). PCR products were purified by Qiaquick gel extraction kit (QIAGEN India Pvt. Ltd, Hilden, Germany) and sequenced using the same primers utilized in PCR. Sequencing PCR was performed using forward and reverse primers (separately) and Big Dye v3.1 dye terminator on ABI Prism 3100 Genetic Analyzer (Applied Biosystems, Foster City, CA) [13]. Number of samples analysed for each target gene were mentioned in Table 1.

**Table 1:** Diversity estimates of *Pfrhs* based on sequence variants in parasite isolates

Gene name	*Pfrh1*	*Pfrh2a/b*	*Pfrh4*	*Pfrh5*
No of samples (n)	47	43	46	83
Length sequenced (bp)	999	1092	921	1320
Polymorphic sites	4	8	6	18
Polymorphic Sites with frequency < 5%	2	7	4	14
Parsimony informative sites	4	2	2	8
No of haplotypes (H)	5	7	7	15
Haplotype diversity (Hd ± SD)	0.660 ± 0.052	0.532 ± 0.074	0.527 ± 0.071	0.594 ± 0.061
Average number of pairwise differences (k)	0.809	0.802	0.995	1.234
Raggedness index	0.182	0.108	0.482	0.055
Recombination event	0	0	0	2
Nucleotide diversity (π ± SD)	0.0008 ± 0.0001	0.0007 ± 0.0002	0.0011 ± 0.0002	0.0002 ± 0.0002
Watterson’s θ ± SD	0.0009 ± 0.0004	0.0017 ± 0.0006	0.0015 ± 0.00061	0.0027 ± 0.0006
Tajima’s D	−0.246	−1.592	−0.702	−1.922^a^
Fu & Li’s D^a^	1.011	−2.837*	−2.204	−2.915^*^
Fu & Li’s F^a^	0.731	−2.866*	−1.912	−3.042^*^
Average number of non-synonymous sites, dN (SE)	0.0007 ± 0.00002	0.0009 ± 0.000041	0.0006 ± 0.00002	0.001 ± 0.00002
Average number of synonymous sites, dS (SE)	0.0011 ± 0.00006	0	0.003 ± 0.0001	0.0002 ± 0.00001
dN/dS	0.7	–	0.2	5.8

^a^indicates p < 0.05

### Sequence alignment and data analysis of PfRH family members

Raw sequence data files from field isolates were manually revised to exclude indefinite reads and signal noise. To compare the sequence identity, NCBI BLAST analysis was performed with all test sequences with respect to reference sequences (Gene ID of *Pfrh1*: PF3D7_0402300; *Pfrh2a*: PF3D7_1335400; *Pf*RH2b: PF3D7_1335300; *Pfrh4*: PF3D7_0424200; *Pfrh5*: PF3D7_0424100) [21]. MEGA 7 tool was used to perform multiple sequence alignment of the nucleotide sequences and to translate them into amino acid codes. Nucleotide sequences analysed in this study were submitted to the GenBank database under the accession numbers MN937274-MN937320, MN950796-MN950967.

Variants were identified by aligning the sequence reads with corresponding reference sequences. Varied number of single nucleotide polymorphisms (SNPs) was observed in both study zones. Genetic diversity parameters including (i) number of segregating (S) sites, (ii) average number of pairwise nucleotide differences within population (k), (iii) average number of observed nucleotide differences per site between any two sequences believing that the sample was randomly selected (π), (iv) Watterson’s θ (θ_w_) were estimated using DnaSPv6.00 [22, 23]. The estimation of the recombination phenomena represented by the minimum number of recombination events (Rm) that happened along the isolates was also performed. Tajima’s D and Fu & Li’s statistics were deployed using DnaSP to assess the neutral theory of evolution [24–26]. To gauge the impact of natural selection over gene sequences the ratio of non-synonymous to synonymous substitutions (dN/dS) is commonly used [27]. The method of Nei and Gojobori with the Jukes and Cantor correction was used to assess these rates of substitution within species as implemented in the MEGA7 [28, 29]. Five hundred bootstrap replications were applied to estimate p values as well as standard error. To derive an estimate of how did parasite sequence data generated in this study differ from those available in global data, particularly those from sub-Saharan Africa, *Pfrh* sequences were retrieved from NCBI and diversity analysis was performed (Additional file 2: Table S2). Further, the evolutionary connections among nucleotide haplotypes of each *Pfrh* family members were predicted using Network program [27, 30–33].

### Ligand receptor interaction: an in silico analysis

To analyse the impact of genetic variability on ligand-receptor interaction efficacy, the most prevalent PfRH5 haplotype (rh5h1) was selected to compare with its 3D7 type counterpart (PfRH5_3D7_). C203 residue in PfRH5_3D7_ was replaced by Y203 in the most prevalent PfRH5 haplotype rh5h1. 4u0q.pdb protein file representing complex of rh5h1 (hereafter, mentioned as PfRH5_M_) with its erythrocyte surface receptor BSG was extracted from RCSB and the interaction efficiency was compared with that of PfRH5_3D7_ and BSG [34]. In order to introduce C203 residue, the side-chain of residue number 203 (Y) of ligand was removed from the 4u0q.pdb file and the residue name of the backbone parts was changed from Y to C. Thereafter, AUTODOCK tool was applied to construct the side chains of the newly modified residue [35]. The pdb file of the PfRH5_3D7_ - BSG complex was thus generated. Both the interaction complexes were refined by minimizing their energies separately in equal number of steps until the difference in the energy between the last two steps is lower than 10% of the preceding step. Afterwards, the effect of the mutation was studied by comparing the residue-wise interaction patterns in the two energy minimized complexes and the H-bonding patterns of two (C/Y) residues at 203 in those structures.

To compute the residue-wise interaction pattern between ligand and receptor, partial atomic charges were assigned to all the atoms of the both complexes using CHARMM. Interaction of the atoms of individual residues in ligand with whole BSG was computed considering two components (i) electrostatic interaction and (ii) van der Waals interaction. Residue-wise interaction in the two complexes and the H-bonding patterns of the residue 203 in the two complexes were also studied.

### Expression of recombinant proteins

To validate the result obtained from in silico analysis, the binding interaction of PfRH5 with BSG was studied in further details. A 564-bp fragment of the *Pfrh5* gene encoding the 188 aa (Glutamine-35—Serine-222 residues) which encompasses receptor binding site, was PCR amplified from DNA of a *P. falciparum* infected blood sample possessing PfRH5_M_ type sequence using forward primer: **CGCGGATCCC**CAAGAAAATAATCTGAC and reverse primer: ACATATGACAAAGTGAAAAGT**AAGCTTGCG** [36]. Polymerase chain reaction was carried out in 15μL reaction mixtures having 1 U of GoTaq^®^ Flexi DNA polymerase (Promega), 0.2 mM dNTP, 1.5 mM MgCl_2_ and 0.4 μM of each primer. The cycling conditions for PCR consisted of an initial denaturation at 94° C for 5 min, followed by 38 cycles of denaturation at 94° C for 45 s, annealing at 58° C for 45 s, extension at 72° C for 45 s, and a final extension at 72° C for 5 min. PCR product was visualized using UV transillumination on gel documentation system (Biostep) following electrophoresis on 2% agarose gel (Promega) and were purified by Qiaquick gel extraction kit (QIAGEN India Pvt. Ltd, Hilden, Germany). The PCR amplicon was digested with *Bam*H I and *Hin*d III (New England Biolabs, Beverly, MA) and inserted downstream of the T7 promoter in the *E. coli* expression vector, pET-20b(+) (Novagen, San Diego, CA) using a T4 DNA ligase (Fermentas), to obtain the plasmid rPfRH5_M_- pET-20b. Site-directed mutagenesis (SDM) was performed on the plasmid rPfRH5_M_- pET-20b to insert C in exchange of Y at 203 amino acid position to generate rPfRH5_3D7_- pET-20b using the SDM-specific primer SDMF: 5′-GTCCTCTACATATGGAAAGTGTATAGC-3′ and SDMR: 5′-AGCATCTACAGCTATACACTTTCCAT-3′. The transcribed sequence of the recombinant plasmids (rPfRH5_3D7_- pET-20b and rPfRH5_M_- pET-20b) contained an additional His-tag (LEHHHHHH) at the C terminus. *E. coli* BL21 (DE3) cells (Novagen, San Diego, CA) were transformed with rPfRH5_3D7_- pET-20b and rPfRH5_M_- pET-20b and used for the expression of rPfRH5_3D7_ and rPfRH5_M_. Sequencing of the plasmid chimera was used to confirm that two different *PfRH5* alleles were inserted in correct reading frame.

Gene expression was performed in a 100 ml culture, using Luria–Bertani medium containing 100 mM ampicillin at 37 °C. Pilot experiments determined that 150 ml bacterial culture incubated for 16 h at 37 °C gave optimal expression of recombinant parasite proteins in culture medium. Once the optical density at 600 nm reached 0.6, the culture was incubated overnight at shaking condition (160 rpm) at 37 ^°^C for protein expression. Cells were harvested by centrifugation next day and the cell pellet was stored at −80 °C. The recombinant protein of the expected molecular mass (24.5 kDa) was present in a total cell lysate.

### Purification of His-tagged recombinant protein by Ni–NTA affinity chromatography

The frozen cell pellet was resuspended in 800 ml of lysis buffer (50 mM NaH_2_PO_4_, 300 mM NaCl, 10 mM imidazole (Qiagen); pH 8.0), mixed at 4 °C for 1 h, and lysed by ultrasound technology (UP200S Ultrasonic Processor; 0.7 cycle; 80% Amplitude), followed by centrifugation at 12,000 rpm for 20 min. Supernatant containing protein was applied on 133 µl of nickel Ni–NTA resin (Qiagen) equilibrated with lysis buffer. To remove non-specifically bound proteins, 1 ml of the wash buffer (pH 7) containing 50 mM imidazole was used. Bound proteins were eluted with elution buffer containing 500 mM imidazole. Eluted fractions were analysed by SDS-PAGE and Western blotting. Concentrations of the fractions containing recombinant PfRH5 was determined by Nanodrop (Thermo Scientific) and stored at −80 °C.

### SDS-PAGE and Western blotting

Crude lysates as well as purified proteins were separated in the presence of SDS using 15% polyacrylamide gel and visualized with Coomassie^®^ Brilliant blue R 250 (Merck). Approximately, 30-50 ug/lane purified protein was electrophoresed by SDS-PAGE using Prism Ultra Protein Ladder (ab116028, abcam; 10–245 kDa) and transferred to polyvinylidene fluoride membrane (Pall Corporation, Port Washington, USA). Blots were treated with primary (mouse monoclonal antibody directed against 6X His tag; ab18184, Abcam) and secondary antibodies and incubated with elctrochemiluminescence (ECL) reagent (SuperSignal West Pico Chemiluminescent Substrate, Thermo Scientific) and examined on Bio-Rad-Molecular imager ChemiDoc™ XRSt with ImageLab^TM^ software.

### Circular dichroism analysis, Fourier transform infrared spectroscopy (FTIR), synchronous fluorescence and fluorescence anisotropy

The folding state of the purified proteins was evaluated by circular dichroism (CD) spectroscopy. CD spectra were recorded on a J-815 CD Spectrometer (Jasco) at 25 °C with the path length of 1 mm. The protein sample solution was diluted in PBS (pH 7.4) so that a final protein concentration was 2 μM. Secondary structural changes were recorded in the range of 190–260 nm. Each spectrum represented an average of three scans and CD data were expressed as mean residue molar ellipticity [θr].

FTIR was carried out using PerkinElmer spectrum 100 FT-IR Spectrometer at a resolution of 0.5 cm^−1^ in the wavenumber range 1000–4000 cm^−1^. 10 ul of solution containing 1ug/ul protein was utilized for each FTIR analysis.

Synchronous fluorescence patterns of PfRH5_3D7_, PfRH5_M_, PfRH5_3D7_–BSG and PfRH5_M_–BSG were observed in different Δλ at 25 °C [37, 38]. Since, in synchronous fluorescence, maximum peak intensity was shown at 379 nm while Δλ was 70, fluorescence anisotropy of BSG, PfRH5_3D7_, PfRH5_M_, PfRH5_3D7_ –BSG (1:1), and PfRH5_M_–BSG (1:1) were performed in the range between 309 and 379 nm (Δλ = 70) for 300 s with both excitation and emission bandwidth of 5 nm using a Varian Cary Eclipse (USA) spectrofluorimeter [39].

### Isothermal titration calorimetry

Isothermal calorimetry is a quantitative tool used to evaluate thermodynamic profile of interaction between two or more molecules in solution, while finding out equilibrium binding constant (K_a_) [40]. In this study, BSG was added drop by drop in a fixed volume with 1 min time interval in the sample cell containing either PfRH5_3D7_ or PfRH5_M_ in a VM2: Cell - ITC 200 (micro cal) titration micro-calorimeter. Concentration of BSG was 10 times higher than PfRH5. Calorimetric reference cell was loaded with 200 μl of Dulbecco phosphate buffer saline solution (pH 7.4). Titration curve for PBS-BSG interaction was subtracted from the heat of binding reaction of PfRH5–BSG to obtain the effective heat of binding. The resulting titration curves were fitted for one set of binding site using MicroCal Origin software.

## Results

### Sequence diversity of genes encoding PfRH ligands

To infer the nature of the evolutionary forces shaping the genetic landscape of *Pfrh* members, fragments encoding *Pfrh1*, *Pfrh2a*, *Pfrh2b*, *Pfrh4* and *Pfrh5* were amplified from genomic DNA isolated from peripheral blood samples of malaria patients. Overlapping sequences from each sample were assembled before estimating the genetic diversity parameters.

#### *Pfrh1*

**Fig. 2:**
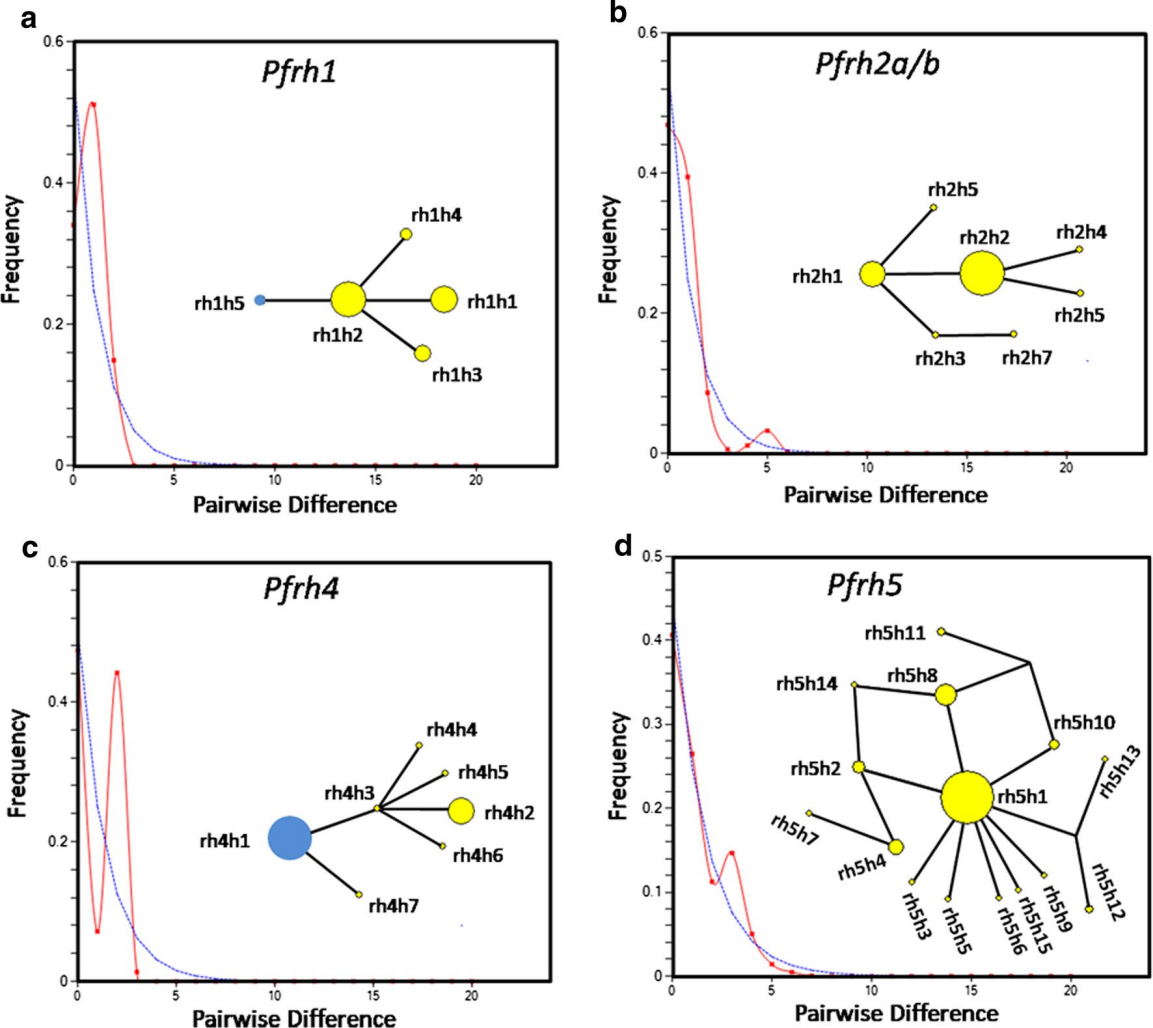
Pairwise mismatch distribution and median-joining network of *Pfrhs*. Graphical representation of pairwise mismatch distribution (**a** *Pfrh1*, **b** *Pfrh2a/b*, **c** *Pfrh4*, **d** *Pfrh5*). Blue line indicated distribution pattern expected under the model of constant population size and red line demonstrated distribution observed in *P. falciparum* population under study. The genetic relationship among the *Pfrh* haplotypes was demonstrated in the insets. Size of a circle was proportional to relative frequency of respective haplotype. Yellow circles represented the extant parasite haplotypes while the blue one indicated the Pf3D7 allele. Each brunch of the network represented a mutational step

Analysis of 1 kb DNA sequences of *Pfrh1* that corresponded to the receptor binding region (500 to 832 amino acids as per PlasmoDB) from 47 malaria patients (15 from Chhattisgarh and 32 from West Bengal) identified 4 Parsimony informative sites of which 2 displayed a frequency > 5%. This resulted in an overall low genetic diversity (Hd = 0.660 ± 0.052; Watterson’s θ = 0.0009 ± 0.0005 and π = 0.0008 ± 0.0001) for the locus. Analysis of median-joining network revealed that a single parasite haplotype (rh1h2) different from the 3D7 type variant (rh1h5) dispersed in the extant population. The demographic history inferred from mismatch distribution pattern revealed no evidence for historical population size fluctuation or recent population expansion (Table 1, Fig. 2a).

#### *Pfrh2a/b*

Genes encoding PfRH2a and PfRH2b shared a common ectodomain sequence that translated into a region spanning 495 to 860 amino acids which was responsible for binding to the receptor. DNA samples from 43 malaria patients (21 from Chhattisgarh and 22 from West Bengal) were employed for the analysis. Unlike other *Pfrh*s, 4 indel polymorphisms with frequencies ranging from 0.023 to 0.81 were detected in addition to 8 single base alterations. Only 2 of these 8 segregating sites were Parsimony informative and 7 had frequencies < 5%. Despite the predominance of low-frequency polymorphisms that yielded negative Tajima’s D (−1.59174) and Fu & Li’s D* and F* statistics (Table 1), the observed mismatch distribution did not deviate from the one expected under demographic expansion (Fig. 2b). Median-joining network analysis disclosed that all of the observed *Pfrh2a/b* haplotypes were evolved from the two most frequent haplotypes namely rh2h1 and rh2h2. The extant parasite population lacked any haplotypes corresponding to Pf3D7 sequence (Fig. 2b).

#### *Pfrh4*

Sequence data representing 282 to 588 amino acid region of *Pf*RH4 were generated using 46 malaria samples (18 from Chhattisgarh and 28 from West Bengal). Application of DnaSP6 resulted in 6 segregating sites and 7 haplotypes (Hd = 0.527 ± 0.071). The most predominant haplotype, rh4h1, resembled *Pf*3D7 allele and was present in both study regions. Presence of only 2 Parsimony informative sites and an excess of low frequency (4 with frequency < 5%) variants yielded a negative Tajima’s D and Fu & Li’s statistics (Table 1). The bimodal mismatch distribution (raggedness index = 0.4819) persisted even when the analysis was replicated using sequences from individual study region and was caused by coexistence of multiple high-frequency parasite haplotypes (Fig. 2c). Network analysis showed that *Pf*3D7 (rh4h1) to be the most predominant allele (Frequency = 0.65) followed by rh4h2 (Frequency = 0.24) (Fig. 2c).

#### *Pfrh5*

Genomic DNAs isolated from blood samples of 83 malaria patients (21 from Chhattisgarh and 62 from West Bengal) were used to analyse the diversity of *Pfrh5* locus. Evaluation of a 1320 bp sequence covering 45 to 484 amino acid region of *Pf*RH5 revealed presence of 18 segregating sites giving rise to 15 haplotypes (Hd = 0.594 ± 0.061). Fourteen segregating sites had a frequency < 5% yielding a statistically significant negative Tajima’s D −1.92225) and Fu & Li’s D* (−2.91441) and F* (−3.04218) statistics (Table 1). The large excess of singletons (n = 10) produced a star-like median-joining network of *Pf*RH5 haplotypes emanating from rh5h1 possessing a single non-synonymous variation (G > A at 815 nucleotide positon) (Fig. 2d). The observed pattern of mismatch distribution adhered strongly to the standard neutral model of panmictic population with constant size (raggedness index = 0.0698), (Fig. 2d). Seventeen out of 18 sites were non-synonymous in character yielding a very high dN/dS ratio of 5.8. However the application of McDonald–Kreitman (MK) test using *P. falciparum* and *P. gaboni* (retrieved from PlasmoDB) data showed a significantly higher (χ^2^ = 4.072; p = 0.043) ratio of nonsynonymous to synonymous amino acid variation within species (17:1) compared to that between species (62:24). This together with the observed reduction of the nucleotide diversity reinstated that purifying selection influenced the currently observed genetic architecture of PfRH5. Interestingly 13 out of 15 haplotypes possessed a the variation that corresponded to C203Y non-synonymous change on Pf3D7 background. Whether this polymorphism offered any gain-of-function from the angle of *Pf*RH5-BSG interaction was next investigated.

#### Comparison of the *Pfrh* sequences

A comparison of the sequence data generated in this study was carried out by using sequences from central India, Papua New Guinea, Kenya and Mali (Additional file 2: Table S2.). Despite the excess of low frequency sites, a statistically significant genetic differentiation (F_ST_) was observed for majority of the loci and geographical regions analysed (Additional file 2: Table S2.). Analysis of *Pfrh5* sequences from Kenya and Mali revealed a higher abundance of C203Y (0.9 and 0.67, respectively) variation as observed in data from Chhattisgarh and West Bengal of the present study (Additional file 3: Fig. S1.) [41–44].

### In silico evaluation of C203Y variation on PfRH5-BSG interaction

**Fig. 3:**
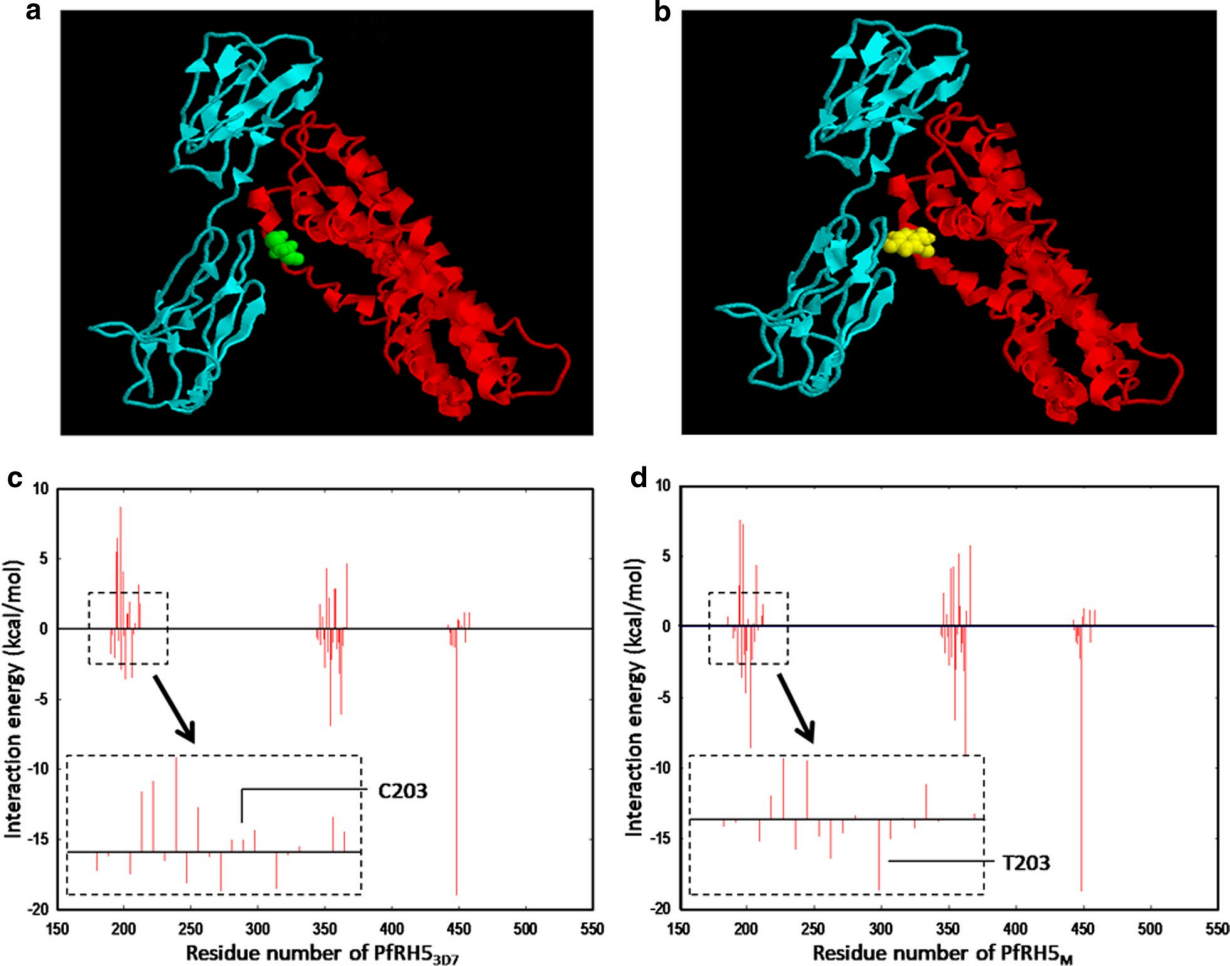
In *silico* modelling of interaction between PfRH5_3D7_ or PfRH5_M_ with BSG. **a** Three dimensional space filled model of the PfRH5_3D7_ (red) and BSG (cyan) complex was shown with the residue C203 highlighted with green color. **b** A model of the PfRH5_3D7-_BSG complex with Y203 was highlighted in yellow was also shown. Energy of interactions (kcal/mol) between BSG and **c** PfRH5_3D7_ or **d** PfRH5_M_ was plotted for each amino acid residue of PfRH5 ligand. The differential interaction patterns around amino acid position 203 for both alleles (C/Y) were indicated (inset)

To understand the exact role of C203Y, a ubiquitously observed nonsynonymous variation, the efficacy of interaction of BSG with PfRH5 encoded by the most prevalent (frequency = 0.63) haplotype, rh5h1 (hereafter mentioned as rPfRH5_M_) was compared to that encoded by the reference Pf3D7 allele (rPfRH5_3D7_). Protein file, 4u0q.pdb, representing a complex of rPfRH5_M_ with BSG was extracted from RCSB protein data bank. A pdb file containing only rPfRH5_M_ was produced using AUTODOCK followed by generation of rPfRH5_3D7_ pdb from the one containing rPfRH5_M_. Ribbon models constructed using pdb files of rPfRH5_3D7_-BSG and rPfRH5_M_-BSG complexes indicated that the Y203 was closer to BSG than C203 (Fig. 3a, b). The interaction efficiencies of two PfRH5 variants with BSG estimated using CHARMM showed that the presence of Y203 resulted in a lowering of total self-energy of rPfRH5_M_-BSG complex (-11200 kcal/mol) compared to that of rPfRH5_3D7_ -BSG (−11127 kcal/mol) indicating that the former was a more stable complex. This was validated by estimation of interaction energy of individual residues of rPfRH5_3D7_ and rPfRH5_M_ in the energy minimized complexes of rPfRH5_M_/rPfRH5_3D7_ with BSG (−10.9 kcal/mol for PfRH5_3D7_ -BSG and -29.9 kcal/mol for PfRH5_M_ –BSG) (Fig. 3c, d). Residue-wise interaction energies varied not only at amino acid position, 203, but in neighbouring positions as well. This signified different conformational arrangement of rPfRH5_3D7_ and rPfRH5_M_ at the time of interaction with BSG (Fig. 3c, d).

### Purification of soluble, biochemically active recombinant PfRH5 proteins

**Fig. 4:**
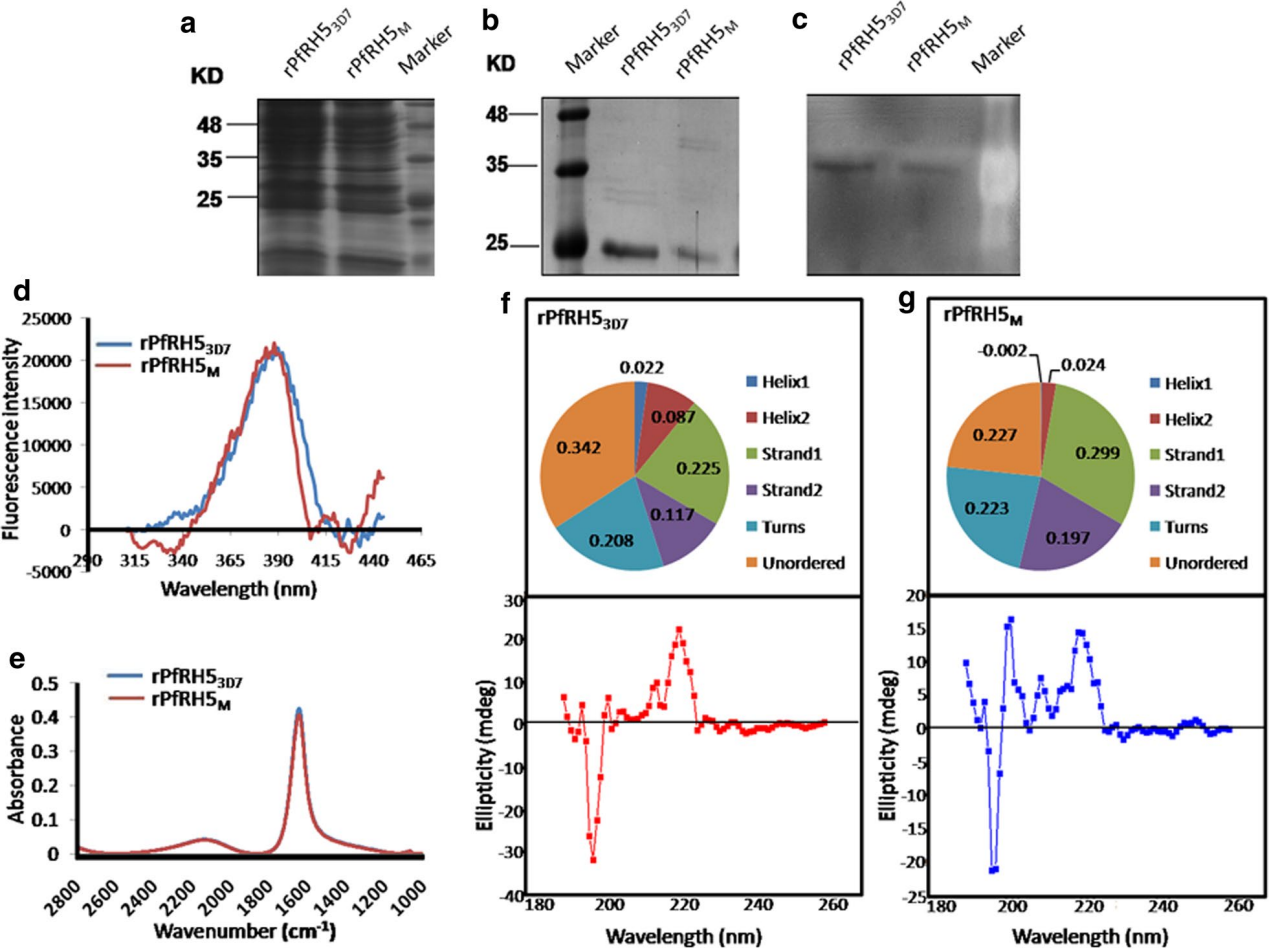
Expression, purification and conformational analysis of the recombinant PfRH5 proteins. **a** Crude cell lysate was stained with Coomassie blue. **b** Representative image of SDS–PAGE in which fractions from Ni–NTA column eluted with 500 mM imidazole was shown. **c** Western blotting of whole cell lysate expressing 6X-His-tag protein. **d** Synchronous fluorescene of rPfRH5_3D7_ and rPfRH5_M_ at Δλ_70_. **e** FTIR spectra of rPfRH5_3D7_ and rPfRH5_M_. Analysis of secondary structure of **f** rPfRH5_3D7_ and **g** rPfRH5_M_ by Far-UV CD spectra (estimated proportions of secondary structural constituents were illustrated in the pie-charts)

DNA sequence representing the receptor binding region of PfRH5 which spanned amino acids 141 to 756 of the protein and harboured Y203 (rPfRH5_M_) allele was PCR amplified from genomic DNA isolated from one *P. falciparum* infected blood sample. The PCR product was cloned downstream to T7 promoter in the *E. coli* expression vector pET-20b (+) in the correct reading frame. C203 (rPfRH5_3D7_) allele was obtained by site-directed mutagenesis using the Y203 containing clone. The chimeric plasmids were mentioned as rPfRH5_3D7_- pET-20b and rPfRH5_M_- pET-20b. Recombinant proteins (rPfRH5_3D7_ and rPfRH5_M_) of expected molecular mass (24.5 kDa) detected in cell lysates were purified by one-step affinity chromatography on Ni–NTA resin (Fig. 4a). Fractions eluted with 500 mM imidazole were separated by SDS-PAGE and detected by Coomassie staining and Western blotting using anti-His tagged antibody (Fig. 4b, c). Yields of purified PfRH5 proteins were 1 mg/ml of *E. coli* culture.

### Conformation analysis of rPfRH5 proteins

Synchronous fluorescence of rPfRH5_3D7_ and rPfRH5_M_ were measured at a range of wavelength differences (Δλ) (Additional file 4: Fig. S2.). Synchronous fluorescence of both proteins showed peak with maximum intensity at Δλ_70_. The spectral pattern at Δλ_70_ did not show any differences between the two variants (Fig. 4d). FTIR spectra of rPfRH5_3D7_ and rPfRH5_M_ also did not differ from each other (Fig. 4e). Variation in folding states of purified rPfRH5 proteins detected as CD data were analysed by SELCON3 programme in Dichroweb (Fig. 4f, g) [45]. CD spectra of rPfRH5_M_ showed an increase in the proportion beta-sheet (49.6% vs 34.2%) at the expense of alpha-helix (2% *vs* 10.9%) and random coil (22.7% *vs* 34.2%) compared to that of rPfRH5_3D7_. Secondary structural components of rPfRH5_M_ differed significantly than that of rPfRH5_3D7_ (p = 0.02) which appeared to be a result of C to Y replacement.

### Characterization of binding affinity of PfRHs with BSG

**Fig. 5:**
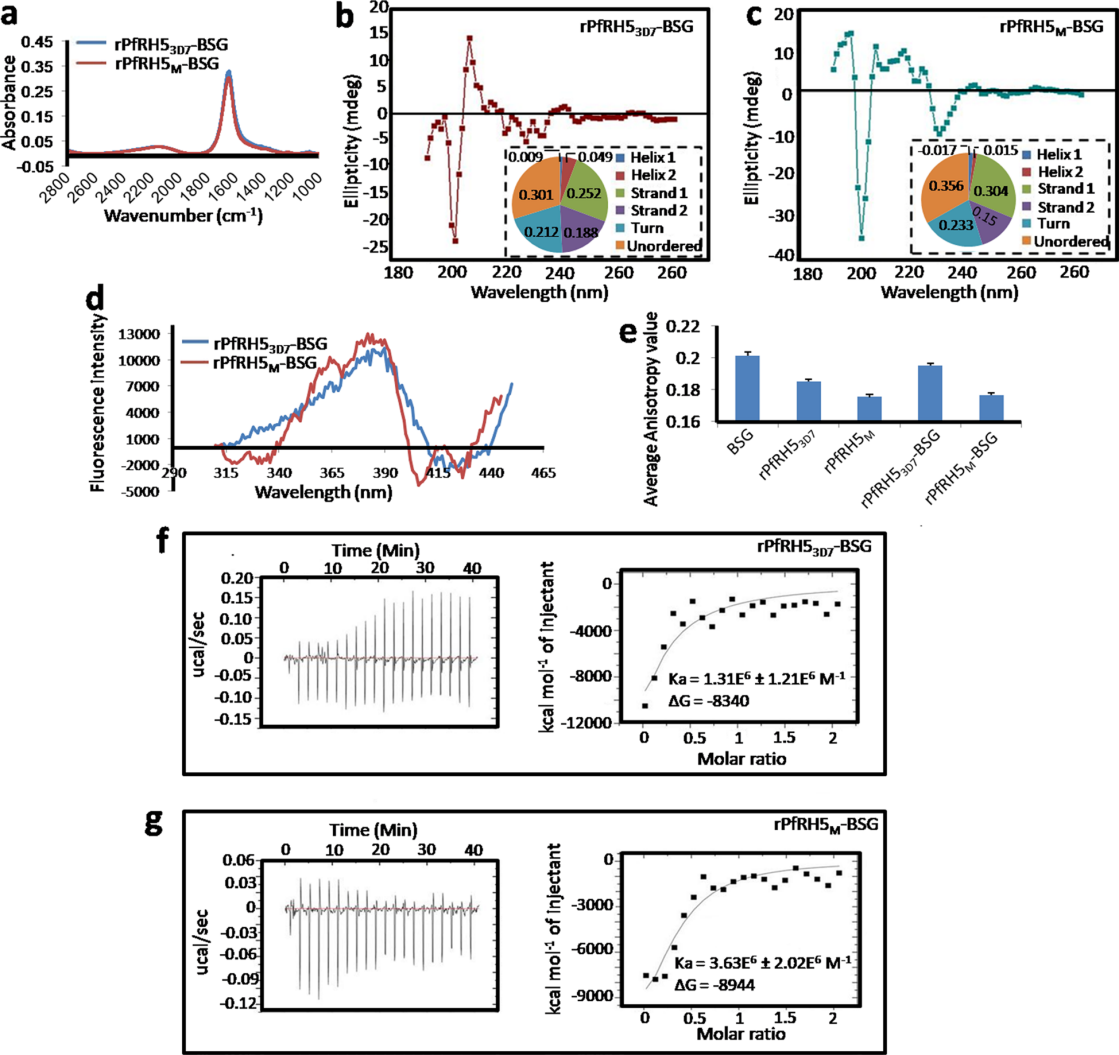
Characterization of binding affinity of PfRH5_3D7_ and PfRH5_M_ with BSG. **a** FTIR spectra of complexes formed between rPfRH5_3D7_ (blue line) or rPfRH5_M_ (red line) with BSG. CD spectroscopy of **b** rPfRH5_3D7_–BSG and **c** rPfRH5_M_–BSG complexes. Estimated proportion of secondary structural constituents was illustrated in adjoining pie-charts. **d** Synchronous fluorescence of rPfRH5_3D7_–BSG and rPfRH5_M_ –BSG complexes at Δλ_70_. **e** Graphical representation of fluorescence anisotropy estimates of receptor (BSG), ligands (RH53D7 and RH5M), and their complexes (BSG-RH53D7 and BSG-RH5M). Data from ITC showing exothermic interaction of BSG with **f** rPfRH5_3D7_ and **g** rPfRH5_M_ at pH 7.0. Each peak indicated an injection of BSG onto respective PfRH5-ligand kept in the ITC cell. The area under the peak is proportional to the amount of heat released during the interaction. ITC profile for binding reactions were generated using 0.1 M phosphate buffer at a temp of 25 °C

To explore the conformation of two ligand-receptor complexes, FTIR, synchronous fluorescence, fluorescence anisotropy UV CD spectroscopy were applied. No significant differences in backbone conformation could be identified when PfRH5_3D7_–BSG and rPfRH5_M_–BSG complexes were subjected to FTIR spectroscopy (Fig. 5a). However, contrary to its unbound state, rPfRH5_M_–BSG exhibited a higher fluorescence peak intensity at Δλ_70_, indicating that either Y203 change or exposure of aromatic amino acids in the rPfRH5_M_–BSG complex was responsible for the observed difference (Fig. 5d). Estimated fluorescence anisotropy indicated a more compact conformation of rPfRH5_M_–BSG complex (0.176) compared to rPfRH5_3D7_–BSG (0.198) (Fig. 5e). CD spectra of rPfRH5_3D7_–BSG and rPfRH5_M_–BSG complexes also showed differences in the percentage of beta-sheet and random coil contents of rPfRH5_3D7_–BSG (50.6% beta-sheets, 25.3% random coil) in comparison to rPfRH5_M_ –BSG (43.4% beta-sheet, 33.3% random coil) (Fig. 5b, c).

Finally, isothermal titration calorimetry was employed to compare the thermodynamic aspects of complexes formed between rPfRH5s and BSG. ITC yielded negative heat deflection, indicating the binding between rPfRH5_M_ and BSG to be an exothermic event. The association constant (K_a_) of rPfRH5_M_ with was found to be 2.77 fold higher (Ka = 3.63E^6^ ± 2.02E^6^ M^−1^) than that between rPfRH5_3D7_ and BSG (Ka = 1.31E^6^ ± 1.21E^6^ M^−1^). This validated a distinct functional impact of C203Y polymorphism in relation to ligand-receptor interaction (Fig. 5f, g). Gibbs free energy of binding (ΔG) was also lower for rPfRH5_M_–BSG (−8944) than rPfRH5_3D7_–BSG (− 8340).

## Discussion

More than 50 years of continued research and control initiatives have caused a commendable reduction of malaria incidence and mortality globally in the last 15 years [46]. In keeping with this progress, the World Health Organization (WHO) has compiled a technical strategy for achieving 90% reduction of malaria incidence and mortality with particular emphasis on development of a malaria vaccine with greater than 75% protective efficacy by 2030 [47]. Till date most malaria vaccines are directed against single stage-specific parasite proteins and are often met with limited success due the mutational diversity of parasite antigens and complex developmental stages of *P. falciparum*. For example, RTS,S which is close to obtaining licensure, displays a modest vaccine efficacy in terms of blocking infection [48]. In addition, there always remains a probability that the vaccine efficacy would deteriorate further due to parasite’s genetic adaptability. These underscore the need for an efficacious vaccine decorated with epitopes from multiple parasite proteins preferably representing pre-erythrocytic and erythrocytic stages. Inclusion of the geographically predominant version of epitopes is another essential criterion. Due to extensive polymorphic nature of merozoite proteins such as AMA-1 and MSP-1, focus has currently been shifted to PfRH antigens [27, 49, 50]. Since, India serves as a large and diverse reservoir of *P. falciparum* parasites, the level and pattern of genetic variability of *Pfrh* family of ligands, which take important part in host-parasite interaction during erythrocyte invasion by *P. falciparum*, are thoroughly investigated.

Not all members of PfRH family are essential for parasite propagation, as they can be knocked out without affecting parasite growth and few members of EBA and PfRH families even perform overlapping function [51, 52]. PfRH5-BSG interaction, nevertheless, is crucial for erythrocyte invasion by *P. falciparum* and PfRH5 antibodies have been shown to exhibit strain-transcending invasion inhibition [19, 53]. Thus a detailed functional analysis of one important PfRH5 variant is also performed.

Analysis of receptor binding regions of *Pfrh1*, *Pfrh2a/b*, *Pfrh4* and *Pfrh5* sequences reveals majority of the polymorphic sites (2/4, 7/8, 4/6 and 14/18, respectively) are rare (frequency < 0.05) among the parasite isolates of Chhattisgarh and West Bengal. For each of these loci, the estimate of nucleotide diversity, π is lower than Watterson’s θ, resulting in a negative Tajima’s D, which typically denotes that evolutionary drivers such as recent population expansion and/or purifying selection/selective sweep may reconstruct the genetic landscape of *Pfrh* genes in *P. falciparum* parasites of these study regions. The observed pattern of mismatch distribution rules out possibility of any past demographic processes. Despite the abundance of low frequency variants, a very high d_N_/d_S_ of 5.7 is observed for *Pfrh5,* which suggests a possibility of genetic hitchhiking effect. Even so, a model for selective sweep due to any advantageous amino acid substitutions cannot be invoked, as the locus harbours significantly high ratio of within-species non-synonymous to synonymous amino acid variations compared to between-species divergence (p = 0.0436). It is thus surmized that genetic architecture of *Pfrh* family members has been shaped predominantly by purifying selection. To compensate for the limited sample size, additional sequences for each of *Pfrh* genes retrieved from NCBI were analysed. Despite an overall abundance of low frequency polymorphisms, the structures of the parasite population from different geographical regions showed remarkable genetic differentiation possibly due to factors such as local adaptation and/or random genetic drift. Nevertheless, frequency of C203Y was found to be high in Kenya and Mali, as well [43, 44].

Since, 17 (D53Y, I60K, N143I, Y147H, H148D, S197Y, C203Y, N245K, N246T, E259A, R298I, M304R, Y307N, V371I, I407V, I410N, K429M) out of the 18 *Pfrh5* variants results in amino acid replacements, an attempt has been undertaken to weigh their possible functional values. Since only C203Y is predominantly present among Indian *P. falciparum* strains, the present study evaluates any functional advantages associated with this replacement in relation to the PfRH5′s interaction with BSG. Analyses of circular dichroism data of purified PfRH5 alone or in complex with BSG show significant differences in the composition of alpha-helices, beta-sheets, and random coils due to C203Y replacement. Pattern of intrinsic fluorescence and anisotropy measures for rPfRH5_M_–BSG complex indicate that alteration in secondary structure of rPfRH5_M_ has further affected the three-dimensional folding of ligand-receptor complex. Estimation of interaction energy and thermodynamic association constant rPfRH5_M_ –BSG further establish a favourable biochemical impact of C203Y polymorphism on the affinity between rPfRH5_M_ and BSG. This data however contrasts a recent study conducted in Mali which fails to find any correlations between amino acid substitutions on PfRH5 with malaria risk [43]. An in vivo validation of the present study in animal or cellular model or by setting up an appropriate epidemiological study specifically addressing whether the parasites with C203Y variant are endowed with increased pathogenicity may resolve this ambiguity. It is noteworthy in this regard that none of the PfCSP polymorphisms have been associated with any clinical risks in paired consecutive infections, but RTS,S which targets CSP still shows allele-specific vaccine efficacy [43, 54, 55]. Taken together, the study unequivocally encourages 203Y version of PfRH5 to be chosen as a prospective vaccine target because of its limited variability across globe in addition to the allele specific effect of the polymorphism in PfRH5-BSG interaction to minimize the possibility of vaccine escape.

## Conclusions

The data presented here clearly demonstrate that genetic architecture of *Pfrh*s is dominated by presence of rare variants due to possible action of purifying selection. C203Y polymorphism located on PfRH5 is present in overwhelming proportion in the study population. This C to Y replacement strengthens the interaction between PfRH5 and BSG. The implication of this genetic variant in the context of host-parasite conversation during merozoite invasion needs to be explored further. Nonetheless, present data is sufficiently informative while designing a rational multivalent malaria vaccine with PfRH5 being one of the components.

## Supplementary information

**Additional file 1: Table S1.** Details of oligonucleotide primers used for amplification and sequencing.

**Additional file 2: Table S2.** Comparison of genetic diversity of Pfrh loci in publicly available global data

**Additional file 3: Fig. S1. a** Neighbor joining network of *Pfrh5* haplotypes (excluding the singletons) observed in India, Kenya and Mali. Size of a circle was proportional to relative frequency of respective haplotype and each brunch of the network represented a mutational step. **b** Frequency of the haplotypes in respective regions.

**Additional file 4: Fig. S2.** Synchronous fluorescence data of recombinant target proteins and their complexes with BSG at different Δλs.

## Data Availability

All sequence data are publicly available at NCBI (www.ncbi.nlm.nih.gov).

## Abbreviations

PfRHs: *P. falciparurm* reticulocyte binding protein-like homologs
PCR: Polymerase chain reaction
SNPs: Single nucleotide polymorphisms
CD: Circular dichroism
FTIR: Fourier transform infrared spectroscopy
