# Supplementary material for: Population genetic and biophysical evidences reveal that purifying selection shapes the genetic landscape of Plasmodium falciparum RH ligands in Chhattisgarh and West Bengal, India
Source: Malar J. 2020 Oct 14;19:367. doi: 10.1186/s12936-020-03433-z (PMC7557104; doi:10.1186/s12936-020-03433-z)
Supplement: Supplementary file 1 — Additional file 1: Table S1. Details of oligonucleotide primers used for amplification and sequencing. [file 12936_2020_3433_MOESM1_ESM.docx]

**Additional file 1: Table S1.** Details of oligonucleotide primers used for amplification and sequencing.

| **Target gene** | **Primer ID** | **Primer sequence** | **PCR condition** |
| --- | --- | --- | --- |
| PfRH1 | 1FP1 | 5'- GCTTAGAAACGATTTAAAAGAATATGC-3' | Initial denaturation at 94°C for 5 min followed by 40 cycles where denaturation at 94°C for 45 sec, annealing at 58°C for 45 sec, extension at 72°C for 45 sec; with a final round of extension at 72°C for 5 min. |
|  | 1RP1 | 5'- GGTTTGTATATGATTAAATGGATTGG-3' |  |
|  | 1FP2 | 5'- CTCTTAAAAGATGAAGCACAATTAGAC-3' | Initial denaturation at 94°C for 5 min followed by 40 cycles where denaturation at 94°C for 45 sec, annealing at 60°C for 40 sec, extension at 72°C for 40 sec; with a final round of extension at 72°C for 5 min. |
|  | 1RP2 | 5'- CTACACAATTCTGTATTTTCTTCTTTTC -3' |  |
| PfRH2a/b | 2FP1 | 5'- CAACAGTAATGACTCTATCAGAACA-3' | Initial denaturation at 94°C for 5 min  followed by 40 cycles where denaturation at 94°C for 45 sec, annealing at 57°C for 45 sec, extension at 72°C for 45 sec; with a final round of extension at 72°C for 5 min. |
|  | 2RP1 | 5'- CAGATTCTAATTCTTCAAAACTTCC-3' |  |
|  | 2FP2 | 5'- GATTCTATAACTCAACGAATAGCAG-3' | Initial denaturation at 94°C for 5 min  followed by 40 cycles where denaturation at 94°C for 45 sec, annealing at 58.5°C for 45 sec, extension at 72°C for 45 sec; with a final round of extension at 72°C for 5 min. |
|  | 2RP2 | 5'- GAACTAAAGAAGCAGTATGCATTTC-3' |  |
| PfRH4 | 4F1 | 5'- GTACCTCCGAATTCACCATTTCG-3' | Initial denaturation at 94°C for 5 min  followed by 40 cycles where denaturation at 94°C for 45 sec, annealing at 60°C for 45 sec, extension at 72°C for 45 sec; with a final round of extension at 72°C for 5 min. |
|  | 4R1 | 5'- CGACTTCTAATGCTTTAGGTTTAATG-3' |  |
|  | 4F2 | 5'- TGAAGATCTTATAAACCAACTACAAC-3' | Initial denaturation at 94°C for 5 min  followed by 40 cycles where denaturation at 94°C for 45 sec, annealing at 59°C for 50 sec, extension at 72°C for 50 sec; with a final round of extension at 72°C for 5 min. |
|  | 4R2 | 5'- GGGATATATAATTGAGGGAGTACC-3' |  |
| PfRH5 | 5FP1 | 5'- CGAGGTCATATGACTCTATCAC -3' | Initial denaturation at 94°C for 5 min  followed by 11 cycles where denaturation at 94°C for 30 sec, annealing at gradually decreasing temperature (- 0.5°C for each cycle) from 63°C to 58°C for 45 sec each,  extension at 72°C for 30 sec; for the next 28 cycles, denaturation at 94°C for 30 sec, annealing at 58°C for 45 sec, extension at 72°C for 30 sec with a final round of extension at 72°C for 5 min. |
|  | 5RP1 | 5'- GCTATACACTTTCCATATGTAGAGGAC -3' |  |
|  | 5FP2 | 5'-CGTTACTACCAATAAAGAGCACTGAAG 3' | Same as for the primer PfRH5 F1 and PfRH5 R1; except, the annealing time is 30 sec for each cycle |
|  | 5RP2 | 5'- CGGTTTCATCATCTGTCTCTTC -3' |  |
|  | 5FP3 | 5'- GTGTATAGCTGTAGATGCTTTTA -3' | Initial denaturation at 94°C for 5 min followed by 7 cycles where denaturation at 94°C for 30 sec, annealing at gradually decreasing temperature (- 0.5°C for each cycle) from 63°C to 60°C for 45 sec each,  extension at 72°C for 30 sec; for the next 32 cycles, denaturation at 94°C for 30 sec, annealing at 60°C for 45 sec, extension at 72°C for 30 sec with a final round of extension at 72°C for 5 min. |
|  | 5RP3 | 5'- ATTCATCTTTTTGAAATGTTCTCC -3' |  |
|  | 5FP4 | 5'- GAACGTTTAAAAAGATGATGGATGAA -3' | Same as for the primer PfRH5 F3 and PfRH5 R3 |
|  | 5RP4 | 5'- CCATGTTTTGTCATTTCATTG -3' |  |
