# Supplementary material for: Population genetic and biophysical evidences reveal that purifying selection shapes the genetic landscape of Plasmodium falciparum RH ligands in Chhattisgarh and West Bengal, India
Source: Malar J. 2020 Oct 14;19:367. doi: 10.1186/s12936-020-03433-z (PMC7557104; doi:10.1186/s12936-020-03433-z)
Supplement: Supplementary file 2 — Additional file 2: Table S2. Comparison of genetic diversity of Pfrh loci in publicly available global data [file 12936_2020_3433_MOESM2_ESM.doc]

**Additional file 2: Table S2.** Comparison of genetic diversity of *Pfrh* loci in publicly available global data

| **Parameters** | **PfRH1** | **PfRH2a/b** | **PfRH4** | **PfRH5** |
| --- | --- | --- | --- | --- |
| **Central India** | | | | |
| **nt (bp) & aa sequences covered** | 999 &  500 – 832 | 537 &  284 – 462 | 789  426 – 588 | NA |
| **n (Accession no)** | 4 (KY202724 – KY202727) | 7 (KY202712 – KY202718) | 3  (KY202706 – KY202708) |  |
| **Polymorphic sites** | 3 | 6 | 3 |  |
| **Parsimony informative sites** | 0 | 4 | 0 |  |
| **No of haplotypes** | 4 | 7 | 3 |  |
| **Haplotype diversity** | 1±0.177 | 1±0.076 | 1± 0.272 |  |
| **nt diversity (π )** | 0.0015± 0.0004 | 0.0050± 0.0008 | 0.0025± 0.0009 |  |
| **Watterson’s θ** | 0.0016±0.0009 | 0.005± 0.002 | 0.002± 0.001 |  |
| **Tajima’s D** | -0.754 | 0.452 | -- |  |
| **Fu & Li’s D* & F*** | -0.754, -0.675 | 0.439, 0.481 | -- |  |
| **dN/dS** | -- | -- | 0.11 |  |
| **F_ST_** | 0.163 | 0.478* | -0.2 |  |
| **Kenya** | | | | |
| **nt (bp) & aa sequences covered** | NA | NA | 477 & 430 – 588 | 1266 & 63 – 484 |
| **n (Accession no)** |  |  | 45 (MG023657 – MG023701) | 39 (MG023323 – MG023361) |
| **Polymorphic sites** |  |  | 3 | 7 |
| **Parsimony informative sites** |  |  | 2 | 4 |
| **No of haplotypes** |  |  | 5 | 9 |
| **Haplotype diversity** |  |  | 0.575±0.049 | 0.730± 0.064 |
| **nt diversity (π )** |  |  | 0.0014±0.0002 | 0.0012±0.0002 |
| **Watterson’s θ** |  |  | 0.0014± 0.0008 | 0.0013±0.0005 |
| **Tajima’s D** |  |  | -0.047 | -0.215 |
| **Fu & Li’s D* & F*** |  |  | -0.384, -0.330 | -0.962, -0.855 |
| **dN/dS** |  |  | -- | -- |
| **F_ST_** |  |  | 0.362* | 0.079* |
| **Mali** | | | | |
| **nt (bp) & aa sequences covered** | NA | NA | NA | 1206 & 53-454 |
| **n (Accession no)** |  |  |  | 198 (MG012489 –MG012686) |
| **Polymorphic sites** |  |  |  | 10 |
| **Parsimony informative sites** |  |  |  | 5 |
| **No of haplotypes** |  |  |  | 13 |
| **Haplotype diversity** |  |  |  | 0.577±0.028 |
| **nt diversity (π)** |  |  |  | 0.00061±0.00005 |
| **Watterson’s θ ± SD** |  |  |  | 0.0014±0.0004 |
| **Tajima’s D** |  |  |  | -1.326 |
| **Fu & Li’s D* & F*** |  |  |  | -2.550* & 2.519* |
| **dN/dS** |  |  |  | -- |
| **F_ST_** |  |  |  | 0.123* |
| **Papua New Guinea** | | | | |
| **Length (bp)** | NA | 750 | 921 | NA |
| **n (Accession no)** |  | 18 (HM802471 - NGHM802488) | 12 (HE610489 - HE610500) |  |
| **Sequence covered** |  | 284 – 533 aa | 282 – 588 aa |  |
| **Polymorphic sites** |  | 8 | 1 |  |
| **Parsimony informative sites** |  | 5 | 1 |  |
| **No of haplotypes** |  | 8 | 2 |  |
| **Haplotype diversity** |  | 0.869±0.049 | 0.409±0.133 |  |
| **nt diversity (π )** |  | 0.0031±0.0003 | 0.0004± 0.0001 |  |
| **Watterson’s θ** |  | 0.003±0.001 | 0.0004± 0.0004 |  |
| **Tajima’s D** |  | -0.038 | 0.540 |  |
| **Fu & Li’s D*& F*** |  | -0.294, -0.257 | 0.752, 0.787 |  |
| **dN/dS** |  | 1.5 | -- |  |
| **F_ST_** |  | 0.438* | 0.162* |  |

nt and aa denote nucleotide and amino acid

F_ST_ was estimated with respect to pooled In parasite samples from the present study

NA denotes data not available
