# Supplementary material for: Population genetic and biophysical evidences reveal that purifying selection shapes the genetic landscape of Plasmodium falciparum RH ligands in Chhattisgarh and West Bengal, India
Source: Malar J. 2020 Oct 14;19:367. doi: 10.1186/s12936-020-03433-z (PMC7557104; doi:10.1186/s12936-020-03433-z)
Supplement: Supplementary file 3 — Additional file 3: Fig. S1. a Neighbor joining network of Pfrh5 haplotypes (excluding the singletons) observed in India, Kenya and Mali. Size of a circle was proportional to relative frequency of respective haplotype and each brunch of the network represented a mutational step. b Frequency of the haplotypes in respective regions. [file 12936_2020_3433_MOESM3_ESM.docx]

**
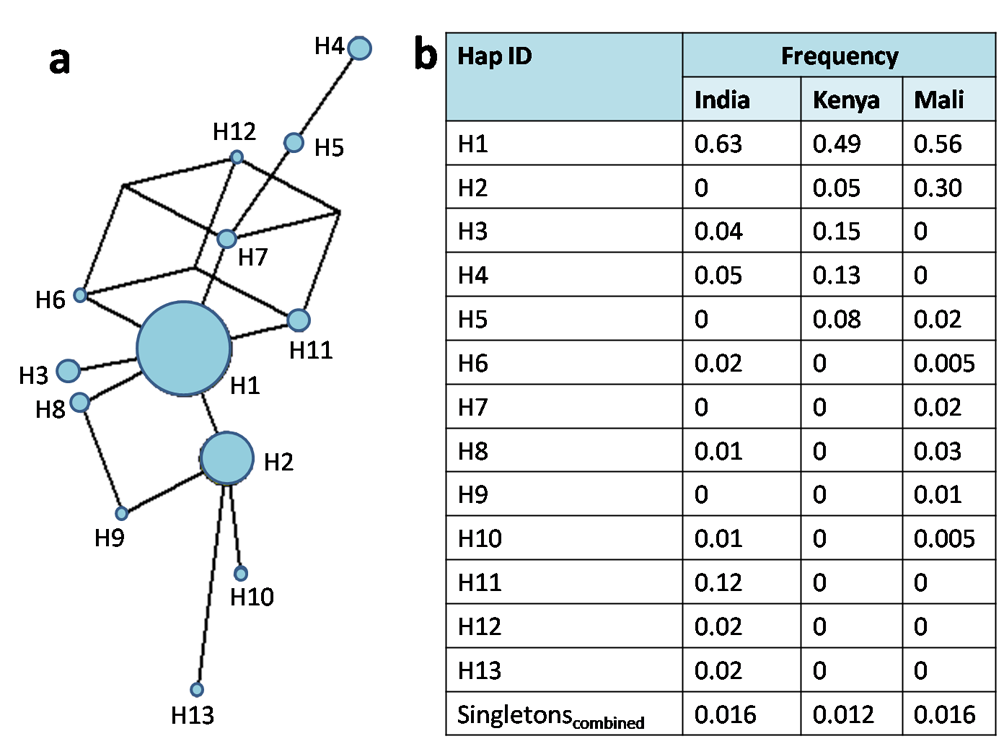
**

**Additional file 3: Fig. S1.** **a** Neighbor joining network of *Pfrh5* haplotypes (excluding the singletons) observed in India, Kenya and Mali. Size of a circle was proportional to relative frequency of respective haplotype and each brunch of the network represented a mutational step. **b** Frequency of the haplotypes used in network analysis.
