# Supplementary material for: Population genetic and biophysical evidences reveal that purifying selection shapes the genetic landscape of Plasmodium falciparum RH ligands in Chhattisgarh and West Bengal, India
Source: Malar J. 2020 Oct 14;19:367. doi: 10.1186/s12936-020-03433-z (PMC7557104; doi:10.1186/s12936-020-03433-z)
Supplement: Supplementary file 4 — Additional file 4: Fig. S2. Synchronous fluorescence data of recombinant target proteins and their complexes with BSG at different Δλs. [file 12936_2020_3433_MOESM4_ESM.docx]

**
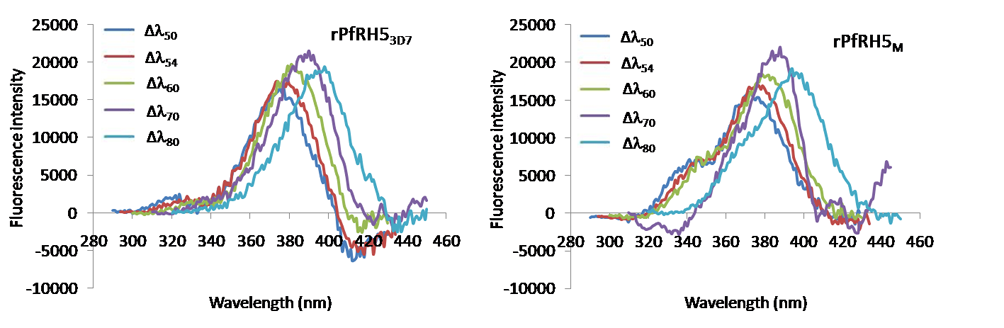
**

**Additional file 2: Fig. S1.** Synchronous fluorescence data of recombinant target proteins and their complexes with BSG at different Δλ.
